# Recent Advances in the Extraction and Characterization of Bioactive Compounds from Corn By-Products

**DOI:** 10.3390/antiox13091142

**Published:** 2024-09-21

**Authors:** Ulises Ramírez-Esparza, María Cristina Agustín-Chávez, Emilio Ochoa-Reyes, Sandra M. Alvarado-González, Leticia X. López-Martínez, Juan A. Ascacio-Valdés, Guillermo C. G. Martínez-Ávila, Lilia Arely Prado-Barragán, José Juan Buenrostro-Figueroa

**Affiliations:** 1 Biotechnology and Bioengineering Laboratory, Research Center in Food and Development, Delicias 33089, Chihuahua, Mexico; uramirez223@estudiantes.ciad.mx (U.R.-E.); magustin123@estudiantes.ciad.mx (M.C.A.-C.); emilio.ochoa@ciad.mx (E.O.-R.); 2 Microbiology and Molecular Biology Laboratory, Research Center in Food and Development, Delicias 33089, Chihuahua, Mexico; salvarado@ciad.mx; 3 CONAHCYT-CIAD, Research Center in Food and Development, Hermosillo 83301, Sonora, Mexico; leticia.lopez@ciad.mx; 4 Bioprocesses and Bioproducts Group, Department of Food Research, Faculty of Chemical Sciences, Universidad Autónoma de Coahuila, Saltillo 25280, Coahuila, Mexico; alberto_ascaciovaldes@uadec.edu.mx; 5 School of Agronomy, Universidad Autónoma de Nuevo León, General Escobedo 66050, Nuevo León, Mexico; guillermo.martinezavl@uanl.edu.mx; 6 Solid Fermentations Pilot Plant, Biotechnology Department, Universidad Autónoma Metropolitana–Iztapalapa, Av. San Rafael Atlixco 186, Col. Vicentina, Ciudad de México 09340, Mexico

**Keywords:** corn colors, phenolic compounds, extraction, biological activities

## Abstract

Maize comes in a variety of colors, including white, yellow, red, blue, and purple, which is due to the presence of phytochemicals such as carotenoids, anthocyanins, flavonoids, phytosterols, and some hydroxycinnamic acid derivatives. In Mexico, maize is primarily grown for human consumption; however, maize residues comprise 51–58% of the total maize plant weight (stalks, leaves, ears, and husks) and are mainly used as livestock feed. These residues contain numerous bioactive compounds that interest the industry for their potential health benefits in preventing or treating degenerative diseases. This review explores the current knowledge and highlights key aspects related to the extraction methods and different techniques for identifying the bioactive compounds found in maize by-products.

## 1. Introduction

Since ancient times, crops have been cultivated worldwide, and countries around the world rely on them for daily sustenance, making the cultivation of maize (*Zea mays* L.) of utmost importance in America [1]. In Mexico, maize is primarily grown for human consumption, representing a crucial source of energy and protein, especially in rural areas and regions with a low socioeconomic status. Millions of Mexicans consume maize daily in various presentations, whether in regional dishes or as tortillas, which is the most important Mexican maize product.

**Figure 1:**
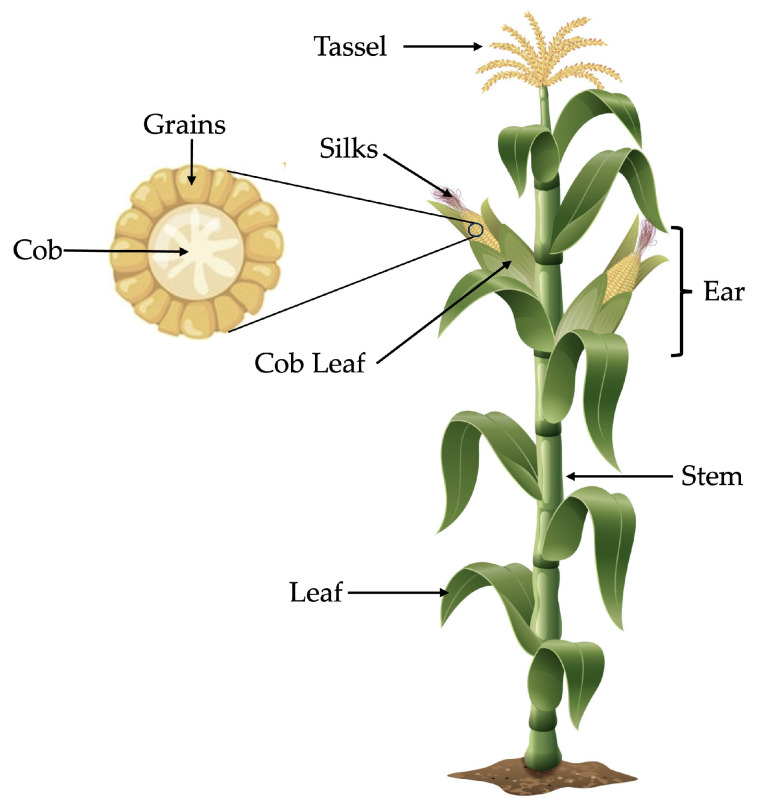
Parts of maize plant.

The maize plant consists of the grain, stem, cob, silks, tassel, and leaves (Figure 1). The maize kernel comprises about 42–49% of the plant’s dry weight [2]. In addition, maize varieties exhibit a range of colors, from white to yellow, red, blue, and purple. These colors are attributed to the presence of phytochemicals such as carotenoids, anthocyanins, flavonoids, phytosterols, and some hydroxycinnamic acid derivatives [3,4,5].

The cob is used as animal feed and for producing bioethanol, oil, biogas, and biocarbon [6], and as a substrate for enzyme production [7]. Similar to the corn kernel, the cob is rich in phenolic acids, anthocyanins, and flavonoids [8,9,10,11].

After maize harvest, the leftover material is called stover, which includes the cob, and it comprises about 51–58% of the plant’s total biomass. Stover consists of stems and leaves, which can be used either green or dry. It is used as livestock feed, and can also be processed to produce biofuels and valuable chemicals like glucan, xylan, and organic acids [12].

This review addresses the general aspects related to the extraction methods and the different techniques for the identification of the bioactive compounds identified in maize by-products, as well as some biological activities that highlight the importance of using corn residues as a valuable source for obtaining these compounds.

## 2. Bioactive Compounds in Maize

The difference between pigmented maize, which can range from red to purple, and conventional maize, usually yellow or white, lies in the presence of anthocyanins. The presence of these compounds contributes to their classification as health-protective foods [13]. Anthocyanins are a type of natural, water-soluble compounds that belong to the group of phenolic compounds known as flavonoids. They consist of glycosides and acylglycosides, which form polyhydroxylated and polymethoxylated heterosides derived from flavylium or 2-phenylbenzopyrilium ions [14]. 

Studies on various maize varieties have identified six major and seventeen minor anthocyanins, including cyanidin-3-glucoside (Cy-3-glu), pelargonidin-3-glucoside (Pg-3-glu), and peonidin-3-glucoside (Pn-3-glu) [5,10,13,15]. Moreover, some varieties, especially purple maize, also contain other flavonoids, such as rutin, hirsutrin, morin, kaempferol, quercetin, naringenin, hesperitin, and their derivatives, which are worth noting [13,16].

Several studies have reported the presence of eight phenolic acids present in various types of maize. These include vanillic acid, syringic acid, 2,4,6-trihydroxybenzoic acid, p-coumaric acid (also known as p-hydroxycinnamic acid), caffeic acid, ferulic acid, chlorogenic acid, and p-hydroxyphenyl acetic acid, and their derivatives. These compounds are typically found in conjugated or bound forms in pigmented maize [11,13,17]. Cuevas Montilla et al. [18] reported that dark maize varieties have higher contents of *p*-coumaric acid and ferulic acids compared to Bolivian yellow ones. In Mexican purple maize, the content of phenolic acids varies among cultivars, with ferulic acid being the most abundant, followed by diferulic and *p*-coumaric acids [19]. Table 1 presents a summary of several studies that have identified different phenolic compounds in diverse maize varieties.

### 2.1. Maize Kernels

In maize kernels, anthocyanins are mainly found in the aleurone and the pericarp. Paulsmeyer et al. [20] reported a greater diversity of anthocyanins in the aleurone layer, although at lower concentrations than those found in the pericarp. However, the variety of pigments in the pericarp and germplasm remains less explored. The pericarp contains flobafenes, which appear as small pigmented lines. These pigments are flavan-4-ols and polymerize to form flavone red pigments, displaying colors ranging from orange to brick red [21]. Maize kernels also contain carotenoids such as lutein, zeaxanthin, β-carotene, β-cryptoxanthin, and α-carotene, particularly in varieties from white to yellow maize [22]. Several studies have demonstrated the antioxidant and anti-diabetic activities of the compounds found in maize kernels [17,23,24,25].

### 2.2. Maize Cob

The maize cob, often considered a by-product in maize processing, is currently underutilized. However, it contains an important amount of bioactive compounds, including anthocyanins and phenolic acids, as detailed in Table 1.

In addition, the maize cob is rich in hemicellulose, suggesting its potential as a source of bioactive oligosaccharides. The unique characteristics of purple maize cob make it an appealing option for extracting compounds that could be used in functional food, cosmetics, and the biomedical industry [26]. In China, anthocyanins extracted from purple maize cob are used as natural colorants in beverages, jellies, and candies [24]. Additionally, maize cob has been reported as a substrate for citric acid production [27]. Natural dyes have also been obtained from pigmented maize cobs [28]. 

### 2.3. Stover

Maize stover consists of the stem, leaf, and husk surrounding the maize. It contains phenolic compounds bound to lignin; lignin has also been reported as a natural antioxidant [29]. Vazquez-Olivo et al. [11] found that maize stover contains total phenols, lignin, as well as specific phenolic acids such as p-coumaric and ferulic acids. Other studies have explored the use of glucose- and xylose-rich stover as a substrate in the fermentation process for producing various organic compounds. These include succinic acid [30], malic acid [31], propionic acid [32], and xylitol [33]. Additionally, phenolic compounds present in maize stover have exhibited biological activities such as anti-inflammatory, neuroprotective, antioxidant, and hepatoprotective properties [34,35,36].

### 2.4. Silk

Maize silk is a by-product that is considered a valuable source of natural bioactive compounds, such as carotenoids, anthocyanins, phenols, alkaloids, saponins, and flavonoids [37,38,39,40]. These compounds are known for their health-promoting effects, which include antioxidant properties, antimicrobial activity, inhibition of lipid peroxidation, analgesic effects, and preventive effects against degenerative diseases [25,41,42,43,44]. 

**Table 1:** Phenolic compounds obtained from the maize plant.

Part	Variety	Group	Compounds	References
Silks	Purple and yellow	Phenolic acids	5-*O*-Caffeoylquinic acid, 3-*O*-Caffeoylquinic acid, 4-*O*-Caffeoylquinic acid, *p*-Coumaroylquinic acid, Maysin and Methoxymaysin derivative	[45]
	Unspecified	Flavonoids	Quercetin, rutin, kaempferol	[46]
	Unspecified		Isoorientin-2-2-*O*-α-L-rhamnoside, 3′-methoxymaysin	[47]
	Unspecified		2″-*O*-α-L-rhamnosyl-6-C-quinovosylluteolin, 2″-*O*-α-L-rhamnosyl6-C-fucosylluteolin, and 2″-*O*-α-L-rhamnosyl-6-C-fucosyl-3′-methoxyluteolin, 2″-*O*-α-L-rhamnosyl-6-C-3″-deoxyglucosyl-3′ methoxyluteolin, 2″-*O*-α-Lrhamnosyl-6-C-(6-deoxyxylo-hexos-4-ulosyl)-luteolin, 2″-*O*-α -L-rhamnosyl6-C-(6-deoxy-xylo-hexos-4-ulosyl)-luteolin-3′-methylether, kaempferol	[38,48]
	Sweet corn		kaempferol-3-*O*-glucoside, luteolin 7-*O*-neohesperidoside, Isoquercitrin, 3′-methoxy maysin, apigenin C-hexose 2″-*O*-deoxyhexoside, apigenin 6-C-deoxyhexose 8-C-pentoside, luteolin *O*-deoxyhexose C-glucuronide and maysin	[49]
Grains	purple	Phenolic acids	Chlorogenic acid, caffeic acid, ferulic acid	[17]
		Flavonoids	Anthocyanins, quercetin, and catechin	
		Carotenoids	lutein, cyclosadol, β-cryptoxanthin, zeaxanthin, α- and β-carotene, α and β-cryptoxanthin	[50]
	purple	Anthocyanins	pelargonidin-3-glucoside, cyanidin-3-glucoside, and peonidin-3-glucoside, cyanidin-3-(6-malonylglucoside), pelargonidin-3-(6-malonylglucoside) and penodin-3-(6-malonylglucoside)	[24]
	Pioneer	Phenolic acids	Ferulic acid and *p*-Coumaric acid	[11]
	Purple	Phenolic acids	Ferulic acid and *p*-Coumaric acid	[4]
	Blue	Anthocyanins	cyanidin 3-glucoside, cyanidin 3-*O*-(6″-succinyl-glucoside), pelargonidin 3-glucoside, pelargonidin 3-*O*-(6″-malonyl-glucoside), cyanidin 3-*O*-(6″-caffeoyl-glucoside) and cyanidin 3-*O*-(600-malonyl-glucoside)	[5]
		Phenolic acids	caffeic acid 4-*O*-hexoside, caffeic acid, 5-*O*-caffeoylquinic acid and *p*-coumaric acid	
		Isoflavone	Daidzin	
		Flavone	apigenin-*O*-hexoside	
	White	Phenolic acids	Gallic acid, Ferulic acid, Protocatechuic acid, *p*-Coumaric acid,	[51]
	Blue	Flavonoids	Catechin	
		Phenolic acids	Ferulic acid, *p*-coumaric acid	
Stem	Dent corn	Phenolic acid derivatives	Methyl (E)-*p*-cumarate, methyl (Z)-*p*-cumarate, methyl ferulate, and 1,3-*O*-diferuloyl glycerol	[34]
Cob	Red	Phenolic acids	Caffeic acid 4-*O*-hexoside, 5-*O*-caffeoylquinic acid, *p*-Coumaric acid	[8]
		Flavonoids	Apigenin-*O*-hexoside, Luteolin-*O*-rutinoside, Apigenin-*O*-pentosyl hexoside, Apigenin 6-C-pentosyl-8-C-hexoside, Procyanidin dimer.	
		Hydroxycumarics	Scopoletin	
	Purple	Anthocyanins	cyanidin-3-glucoside, pelargonidin-3-glucoside, peonidin-3-glucoside, cyanidin-3-(6-malon)-glucoside, pelargonidin-3-(6-malon)-glucoside, peonidin-3-(6-malon)-glucoside.	[9,24]
	Cacahuacintle maize	Anthocyanins	cyanidin-3-glucoside, pelargonidin-3-glucoside, peonidin-3-glucoside, cyanidin-3-(6″malonyl) glucoside, pelargonidin-3-(6″malonyl) glucoside and peonidin-3-(6″malonyl) glucoside	[10]
	Pioneer	Phenolic acids	Ferulic acid and *p*-Coumaric acid	[11]
Cob leaves	Cacahuacintle maize	Anthocyanins	cyanidin-3-glucoside, pelargonidin-3-glucoside, peonidin-3-glucoside, cyanidin-3-(6″malonyl)-glucoside, pelargonidin-3-(6″malonyl)-glucoside and peonidin-3-(6″malonyl)-glucoside	[10]
Stover	Pioneer	Phenolic acids	Ferulic acid and *p*-Coumaric acid	[11]
Tassel	Unspecified	Phenolic acids	Gallic acid, Caffeic acid, Ferulic acid, Syringic acid, Ellagic acid, *p*-Coumaric acid	[52]
		Flavonoid	Rutin, Catechin, Taxifolin	
		Flavanone	Naringenin	
		Flavonol	Kaempferol	
		Other	Methyl gallate, Pyrocatechol	

## 3. Biological Activities of Maize Components

Throughout history, plants and crops containing phenolic compounds have been important in traditional medicine and used by different cultures to treat illnesses and maintain good health. One notable example is the maize kernel. The bioactive compounds found in maize kernels differ depending on the type of maize. Purple maize is rich in anthocyanins, which offer significant health benefits (Table 2). These benefits include antioxidant properties, anti-inflammatory effects [53], cardiovascular protection [54], and anti-diabetic benefits [55]. 

### 3.1. Antioxidant Capacity (In Vitro)

Regarding antioxidant capacity tested in terms of DPPH, ABTS, FRAP, and ORAC, the antioxidant capacity of maize is highly correlated with its contents of various bioactive compounds, including anthocyanins, flavonoids, phenolic acids, polyphenols, and carotenoids. Notably, the phenolic compounds in purple maize have shown higher antioxidant capacities compared to those obtained from other sources, such as cranberry juice [65,66]. Some studies are shown in Table 2.

Additionally, research has shown that the antioxidant levels of Mexican blue and American blue maize remain high even after undergoing industrial processing such as nixtamalization and cooking. Although there is a significant decrease in the anthocyanin content (37 to 75%) and a corresponding reduction in the antioxidant capacity (28–55%), the antioxidant levels remain relatively high [51]. The observed decrease in anthocyanin content and the concomitant antioxidant capacity may be attributed to the degradation of the bioactive compounds during the industrialization process, which involves alkaline and high-temperature processes [67].

### 3.2. Anti-Cancer Activity 

The health benefits of purple maize have been extensively studied using different methods, including in vitro cellular analysis and in vivo animal studies. Anthocyanins also have anti-cancer properties [55] and can inhibit the spread of human colon cancer cells [68] due to their ability to neutralize superoxide radicals [69]. The anti-cancer activity of purple maize has been linked to a combination of anthocyanins, such as cyanidin-3-glucoside, pelargonidin-3-glucoside, and peonidin-3-glucoside. These compounds have been observed to slow the progression of prostate cancer [70] and have effects against HT-29 human colon cancer cells [71,72]. Hagiwara et al. [73] found that extracts from purple maize inhibited the development of colorectal cancer in male rats. Zhang et al. [74] reported protective effects on the liver and kidney of rats. Additionally, Mendoza-Díaz et al. [75] observed antimutagenic activity using the Ames test. Similarly, Reynoso-Camacho et al. [76] found that consuming tortillas made from white, yellow, red, and blue maize provided protection against adenocarcinomas in rats. Specifically, rats that consumed white and blue maize tortillas developed 77.5% fewer tumors, while those consuming red and yellow tortillas showed a 55% reduction in tumor incidence. These studies indicate that, despite the industrialization process, including alkalinization and exposure to high temperatures, maize retains significant anticarcinogenic activity.

### 3.3. Anti-Inflammatory Activity

Another effect of the phenolic compounds present in corn is the ability to provide an anti-inflammatory response. Several studies describe this effect as a great benefit to health. Agrizzi Verediano et al. [77], using an in vivo model (*Gallus gallus*) to analyze the soluble extracts of black corn, showed that these extracts exhibit anti-inflammatory properties due to the decrease in proinflammatory cytokines triggered by the nuclear factor kappa-B (NF-κB) pathway. In other studies, Koraneeyakijkulchai et al. [78] demonstrated that a sweet corn extract can inhibit inflammation in age-related macular degeneration by suppressing the NF-κB signaling pathway.

### 3.4. Other Effects

The residues from processing maize kernels contain bioactive compounds. Vazquez-Olivo et al. [11] found that yellow maize cob, leaf, husk, and stover have antioxidant properties, particularly the husk, which has a high polyphenolic content. Rouf Shah et al. [3] noted that maize silks have been traditionally used in countries like India, China, Spain, France, and Greece to treat kidney stones, urinary tract infections, jaundice, and fluid retention. These therapeutic properties are attributed to the bioactive compounds identified in Table 1 and their antioxidant capacity. There are documented uses of maize silk extracts, and studies in rats suggested protective effects against several diseases, including diuresis and kaliuresis [79], hyperglycemia [80], diabetes [41], nephlotoxicity [81], and inflammatory processes [66]. Additionally, the anthocyanins in purple maize can act as chemopreventive agents, potentially preventing the development of preneoplastic liver lesions [82].

Another effect of the phenolic compounds from corn is antifungal action, which can prevent fungal growth and spore development, as well as avoid the presence of mycotoxins or aflatoxins in corn-derived products [83,84]. Khan et al. [85] obtained corn silk extracts, which showed a favorable antimicrobial effect against several bacteria (*Staphylococcus aureus, Candida albicans, Mycobacterium smegmatis,* and *Escherichia coli*) and presented an inhibitory effect against *Fusarium verticillioides* present in cherry tomatoes. Several studies reported specific antifungal activity for several phenolic compounds, such as ferulic acid and *p*-coumaric acid (present in different parts of corn), demonstrating favorable effects in inhibiting the growth of *Monilinia ructicola, Botrytis cinerea,* and *Alternaria alternata* when using a minimum inhibitory concentration (1.78–3.63 mM) [86]. Lorán et al. [87] demonstrated in their study that various phenolic acids (caffeic, ferulic, and *p*-coumaric) can inhibit aflatoxin production by *Aspergillus parasiticus* at a concentration of 20 mM.

## 4. Extraction, Separation, Identification, and Quantification of Bioactive Compounds from Maize

### 4.1. Extraction

It is essential to carefully optimize the extraction processes for the bioactive compounds from maize to maximize their yields and minimize the changes in the functional properties of the extracted compounds [88]. Maize contains a wide range of phytochemical compounds, including phenolic compounds, carotenoids, and phytosterols. The concentrations of these compounds vary among the different maize varieties [89]. These compounds can be extracted in either free or bound forms depending on the extracting solvents and techniques (Table 3 and Table 4).

The extraction of phytochemicals has been accomplished using water, acetone, alcohols, ethyl acetate, and hexane individually or in combinations (Table 4). For instance, free phenols were extracted using 80% acetone, while bound phenols were extracted by using ethyl acetate after digestion with sodium hydroxide [95]. Hu and Xu [96] used methanol 99% and 1% HCl for carotenoid extraction from maize. Fernandez-Aulis et al. [10] compared different solvents (methanol, ethanol, and acetone in different proportions) for anthocyanin extraction, finding that methanol/water/lactic acid (80:20:1) and ethanol/water/lactic acid (80:19:1) yielded comparable results, while acetone had the lowest yield. Mohsen and Ammar [97] also examined different solvents for maize tassel extraction, determining that ethanol and methanol were the most effective. In addition, Lao and Giusti [9] evaluated various solvents and found that a mixture of ethanol and water (50:50) acidified with 0.01% of 6 N HCl yielded the best extraction of phenolic compounds.

**Table 4:** Some solvents used for the extraction of phenolic compounds.

Parts	Solvents	Reference
Stubble	Ethanol 80%	[11]
Corn kernels	Ethanol 80%	[98]
Yellow corn	Ethanol 80%	[99]
Grains	Ethanol 80%	[100]
Seed and cob	100% Methanol	[24]
Tassel	Ethanol 60%	[52]
Cob	Ethanol in different proportions	[8]
Grains	Methanol acidified with 1 N HCl (85:15, *v*/*v*)	[101]
Kernels	Methanol, Water, and Formic Acid (80:19:1)	[102]
Cobs	Water	[103]
Kernels	Methanol 80%	[104]
Grains	Ethanol 80%	[105]

The traditional solvent-based extraction methods have been widely used. However, there have been reports of unconventional techniques being implemented. These include ultrasound-assisted extraction (UAE), microwave-assisted extraction (MAE), and supercritical fluid extraction (SFE). Additionally, biotechnological approaches such as enzyme-assisted extraction (EAE) and fermentation-assisted extraction (FAE) are gaining attention for their potential to enhance the extraction processes [106]. Table 5 shows the advantages and disadvantages of these unconventional techniques.

Biotechnological methods can be used to release and extract phenolic compounds effectively. This can be accomplished by employing enzyme-assisted extraction (EAE), which breaks down the cell walls, or through a fermentation process in either a liquid or solid medium. During fermentation, microorganisms produce the necessary enzymes to break down the cell walls and transform high-molecular-weight compounds into lower-molecular-weight ones, thus releasing phenolic compounds [107,108]. Solid-state fermentation has been shown to enhance the extraction of polyphenols from various substrates, including gobernadora (*Larrea tridentata*), tarbush *(Flourensia cernua*), Castilla Rose (*Purshia plicata*), pomegranate peel (*Punica granatum* L.), and fig (*Ficus carica* L.) [109,110,111]. Topakas et al. [112] achieved 0.85 g/kg of ferulic acid and 0.38 g/kg of coumaric acid by using combined SSF and EAE from maize cob with *Sporotrichum thermophile* over a 48 h process. In a separate study, Chandra and Arora [113] also utilized maize cob to obtain compounds with antioxidant capacity using various *Aspergillus* strains, resulting in up to a 2.8-fold increase in the antioxidant capacity of the maize cob compared to unfermented material. Acosta-Estrada et al. [114] employed nejayote as a substrate for the growth of *Aspergillus oryzae, Pleurotus ostreatus (Perla and Blue),* and *Hericium erinaceus*, leading to a significant increase in the phenolic content, up to 327% using *Pleurotus ostreatus Perla*. Furthermore, Mahalaxmi et al. [115], using SSF with *Amycolatopsis* sp. RSP 3, successfully obtained rifamycin B from maize husk. Wang et al. [116] developed several methodologies for obtaining D-lactic acid via SSF and EAE processes using maize stover, achieving a yield of 18 g/L with a purity of 99%.

**Table 5:** Advantages and disadvantages of unconventional techniques.

Extraction Method	Advantages	Disadvantages
UAE	Low solvent consumption High extraction fields Short extraction time High reproducibility Low energy consumption	Filtration required Effects of cavitation Difficulty in scaling
MAE	Fast extraction Low solvent consumption High reproducibility Low energy consumption	High equipment cost Filtration required Many parameters to optimize
SFE	Fast extraction Possibility to reuse CO_2_ No filtration required	High equipment cost
EAE	High selectivity Biodegradable High extraction fields	Filtration required Difficulty in scaling High cost of enzymes
FAE	Low prices Biodegradable High extraction fields Low energy consumption Low substrate costs Low cost of process	Contamination Difficulty in scaling The parameters are difficult to control Filtration required

Multiple studies have investigated the use of eco-friendly processes, known as green processes, to extract bioactive compounds from natural sources. For instance, Gullón et al. [26] utilized a hydrothermal method to extract phytochemicals from pigmented maize cob, resulting in a high concentration of bioactive compounds with significant antioxidant properties. Additionally, they identified 15 antioxidant phenolic compounds in the extract. Another study demonstrated that applying ohmic heating to maize flours after a nixtamalization process with low humidity increased the total phenol content compared to the traditional nixtamalization method [117]. Furthermore, the use of high pressures at 700 MPa was found to enhance the total phenol and anthocyanin content in waxy purple maize [118]. The stability of anthocyanins decreases after extraction, and they often remain strongly bound to their original matrix [102]. Because phytochemicals have diverse polarities, it is practically impossible to extract all of them using a single method or solvent. Therefore, selecting the right solvent becomes crucial, aligning with the polarity of the targeted compounds. Additionally, the extraction yield varies depending on factors such as the extraction method used, sequential extraction, and the use of solvents with different polarities [119]. Although anthocyanins are water-soluble, extracting them efficiently often requires a combination with other solvents such as methanol, ethanol, or acetone [95,120].

### 4.2. Separation of Bioactive Compounds

For the separation of bioactive compounds, high-performance liquid chromatography (HPLC) systems are commonly used, either alone or coupled to more advanced systems, such as mass spectrometry (LC–MS). Yang et al. [95] separated phenolic compounds and flavonoids using reversed-phase HPLC (RP-HPLC) with a C18 column, acidified water mobile phase, and acetonitrile. In another study, Hu and Xu [96] used an RP-HPLC system with a diode array detector, C18 column, and a mobile phase composed of acidified water and acetonitrile for the separation of phenols. Carotenoids are separated using an HPLC system equipped with a diode array detector and a C30 column, using methanol and methyl tert-butyl ether as the mobile phase. High-performance thin-layer chromatography plates [121], acid precipitation, Sephadex LH-20 chromatography, filtration [122], microfiltration, and ultrafiltration with membrane [123] techniques have also been used to separate bioactive compounds from a mixed sample. 

### 4.3. Identification and Quantification 

The identification and quantification of the phenolic compounds are performed using commercial reference standards, comparing their retention time and the UV spectrum of the peak or compound of interest. The quantitative data are calculated from a linear calibration curve, elaborated with the standard compound at different concentrations and under the same working conditions of the samples.

There are more sophisticated identification and quantification methodologies, such as Liquid Chromatography–High Resolution Mass Spectrophotometry (LC–HR-MS) and Ultra-High-Performance Liquid Chromatography (UHPLC) coupled to a triple quadruple QToF-MS (time-of-flight), which allow us to have the greatest monitoring of compounds with exact mass measurements.

Another methodology used for the identification of the phenolic compounds in corn is Fourier transform infrared spectroscopy (FT-IR) due to its speed, sensitivity, and easy sample preparation. A methodology that has advanced in recent years is the identification of compounds by nuclear magnetic resonance (NMR) due to the reduced analysis time, high sensitivity, and minimum sample volume required [124]. Table 6 shows some methodologies used for the identification of the bioactive compounds in maize.

## 5. Perspectives and Conclusions

This review highlights the importance of the integral utilization of corn residues to obtain bioactive compounds, thus promoting agricultural sustainability and the development of products of added value in the food and pharmaceutical industries. It is now known that both maize and its by-products (cob, maize hairs, and stover) contain bioactive phenolic compounds, such as phenolic acids, anthocyanins, and other flavonoids. These compounds have demonstrated numerous health-protective properties (antioxidant properties, anti-inflammatory effects, cardiovascular protection, and anti-diabetic benefits), as evidenced by both in vitro and in vivo studies. Most of the research has focused on extracting and characterizing the phenolic compounds present in maize grains. Therefore, there is an opportunity to conduct studies using the complete food matrix or individual phenolic compounds isolated and purified directly from the different parts that comprise maize to revalue these by-products. It has been reported that the phenolic compounds from purple maize are more efficient, but no direct comparison studies were found regarding the efficiency of the different bioactive compounds obtained from the different maize varieties, either in extract form or after undergoing purification processes. There are limited studies aimed at extracting bioactive compounds from maize residues (cob, stubble, and maize silks) using biotechnological processes, such as solid-state fermentation, which has proven to be an effective strategy for proposing alternatives for the use of agro-industrial waste by utilizing microorganisms to add value to these materials in obtaining industrially relevant molecules.

## Figures and Tables

**Table 2:** Bioactivity of phenolic compounds found in maize.

Phenolic Compound	Parts	Effects	Reference
Quercetin	Silks	Antioxidative, anti-inflammatory, anti-proliferative, anti-carcinogenic, anti-diabetic, and anti-viral	[56]
Rutin	Tassel, silks	Anti-diabetic, antioxidant, anti-carcinogenic, anti-allergic, anti-inflammatory	[57]
Ferulic acid	Grains, leaves, tassel	antioxidant, anti-inflammatory, anti-diabetic, anti-depressive	[58]
Cyanidin-3-glucoside	Grains, cob, leaves	anti-inflammatory, anti-cancer, anti-diabetic, anti-toxicity, cardiovascular, and nervous protective capacities	[59]
*p*-Coumaric acid	Grains, cob, stover, tassel	antioxidant, anti-inflammatory, analgesic and anti-antimicrobial properties	[60]
Caffeic acid	Grains, cob, stover, tassel	anti-inflammatory, anti-cancer, anti-diabetic, anti-neurodegenerative diseases	[61]
Catechin	Grains, tassel	anti-inflammatory, anti-cancer and antioxidant	[62]
Pelargonidin-3-glucoside	Grains, cob, leaves	antioxidant, and anti-inflammatory	[63]
Kaempferol-3-*O*-glucoside	Silks, grains, tassel	Anti-carcinogenic and anti-inflammatory	[64]

**Table 3:** Phenolic content and antioxidant capacity in different parts of maize.

Part of the Corn	Solvent	TPC	DPPH	TAC	Reference
Silks	Acetone–water (70:30 *v*/*v*)	2093.9–10,160.8 mg CGAE/100 g		1.49–192.9 mg CGE/100 g	[45]
	Ethanol 70% *v*/*v*		59.20–65.20%		[90]
	Methanol 80%	20.82 mg GAE/g DM	75.65%	42.53 GCG/kg DM	[91]
	Ethanol 95% *v*/*v*	164.1 μg GAE/g	EC50 14.24 μg/mL		
Grains	Ethanol 30% with citric acid 1%	0.33 mg GAE/g	17.72 mg TE/100 g DM		[92]
	Methanol 80% acidified with 1% HCl	9.06 g GAE/kg	EC50 66.3 μg/mL	2.76 CGE/kg	[17]
	Ethanol 25% acidified with 2% formic acid	11.67 g GAE/kg	66.77 μmol TE/g		[93]
	Methanol	–	EC50 48.5 μg/mL	55.8 mg CGE/100 g	[24]
Cob	Ethanol 20% acidified with 1 N HCl	90 mg GAE/g DM		30 mg CGE/g DM	[94]
	Methanol		EC50 40.1 μg/mL	92.3 mg CGE/100 g	[24]
Stubble	Ethanol 80%	933.82 mg GAE/100 g	11.75 mmol TE/g		[11]

TPC: Total phenolic content; TAC: total anthocyanin content; CGAE: chlorogenic acid equivalent; CGE: cyanidin 3-glucoside equivalent; GAE: gallic acid equivalent; TE: Trolox equivalent; DM: dry matter.

**Table 6:** Methodologies commonly used for the identification of bioactive compounds in maize.

Part of the Maize	Methodology	Reference
Silk	FT-IR	[125]
Grains	HPLC	[18]
Maize bran fiber	HPLC–MS, NMR	[126]
Grains	HPLC−QTOF-MS	[127]
Silk	NMR	[85]
Cob	HPLC	[128]
Stover	FT-IR	[129]

## Data Availability

Data will be made available upon request.
